# Assessment of Lumbar Lordosis Distribution with a Novel Mathematical Approach and Its Adaptation for Lumbar Intervertebral Disc Degeneration

**DOI:** 10.1155/2020/7312125

**Published:** 2020-04-15

**Authors:** Zoltan Sandor, Gabor Kristof Rathonyi, Elek Dinya

**Affiliations:** ^1^Institute of Digital Health Sciences, Semmelweis University, Budapest, Hungary; ^2^Department of Orthopaedics, Health Services of Budavari Local Government, Budapest, Hungary

## Abstract

**Introduction:**

Low back pain and disc degeneration could be linked to global spinal geometry. Our study aimed to develop a reliable new mathematical method to assess the local distribution of total lumbar lordosis with a single numeric parameter and compare it with lumbar intervertebral disc degeneration using routine MRI scans.

**Methods:**

An online, open access, easy-to-use platform for measurements was developed based on a novel mathematical approach using MRIs of 60 patients. Our Spinalyze Software can be used online with uploaded MRIs. Several new parameters were introduced and assessed to describe variation in segmental lordosis distribution with a single numerical value. The Pfirrmann grading system was used for the classification of lumbar intervertebral disc degeneration. Relationships were investigated between the grade categories of L1-S1 lumbar discs and the MRI morphological parameters with correlation analysis.

**Results:**

Results confirm that the determination of measurement points and calculated parameters are reliable (ICCs and Pearson *r* values > 0.90), and these parameters were independent of gender. The digression percentage (*K*%), one of our new parameters, did not show a statistical relationship with the Cobb-angle. According to our results, the maximum deflection breaking-point of lumbar lordosis and its location can be different with the same Cobb-angle and the distribution of global lordosis is uneven because the shape of the lumbar lordosis is shifted downward and centered around the L4 lumbar vertebra. The interobserver reliability of the Pfirrmann grades reading was in the excellent agreement category (88.33% agreement percentage, 0.84 kappa), and digression percentage (*K*%) showed a significant negative correlation with all L1-S1 disc grades with increasing *r* correlation values. This means that the smaller the value of digression percentage (*K*%), the more the number of worn discs in the lower lumbar sections.

**Conclusions:**

Spinalyze Software based on a novel mathematical approach provides a free, easy-to-use, reliable, and online measurement tool using standard MRIs to approximate the curvature of lumbar lordosis. The new reliable *K*% (digression percentage) is one single quantitative parameter to assess the local distribution of total lumbar lordosis. The results indicate that digression percentage (*K*%) may possibly be associated with the development of lumbar intervertebral disc degeneration. Further evaluation is needed to assess its behavior and advantage.

## 1. Introduction

Low back pain is a major socioeconomic health issue [1, 2] affecting at least once up to 80% of the entire population [3, 4]. Therapy is complex, no single method has been proven effective [5], and prediction of outcome is not feasible [6, 7]. Factors of sagittal balance, including lumbar lordosis, have been well documented [8–11] and used for surgical planning. However, special imaging is required for the assessment of each factor, and standard, daily practice clinical MRI (magnetic resonance imaging) measures only lumbar lordosis. Total lordosis has not been linked to degenerative changes or pain in a review by Been and Kalichman [12]. In contrast, segmental (local) lumbar spine degenerative changes were linked to pain [13]. One factor might explain this controversy. Lordosis is not evenly distributed and nearly 75% of total lordosis can be accounted for through the lower two (L4-L5 and L5-S1) segments [10, 14] where most disc herniations are observed (up to 95%) [15, 16]. There might be a link between the segmental (local) distribution of overall (global) lordosis and disc degenerative changes.

In the literature, many methods are used to measure and approximate the curvature of lumbar lordosis. There are advantages and disadvantages to each method. The Cobb method (or modified Cobb method) has become the gold standard in the measurement of lumbar lordosis. The Cobb method was one of the first methods used for the measurement of the sagittal spinal curvature. The modified Cobb method was used to evaluate lumbar lordosis as well. In the modified Cobb method, the spine was approximated with an arc. There are also methods to approximate the spine with an ellipse which is a more precise approximation of the spine. Several studies attempted to measure the spinal curvature with mathematical methods; e.g., different functions were used for modelling such as trigonometric functions, splines, and polynomials. The good attributions of the functions were used to give information about the spine, e.g., the area under the curve. There are two thorough reviews of lumbar lordosis and methods for quantitative evaluation of spinal curvature [12, 17].

Distribution and positional change of lumbar lordosis in the normal healthy spine were investigated using an active shape model with MRIs and revealed wide intersubject variation in lumbar spine shape and partial preservation of this shape between postures and movements [18–20]. However, their parameter of segmental distribution of lordosis (mode 2) was only partially able to describe where the curve is uneven without quantifying the location of the apex and only healthy subjects were included. Recently, quantitative fluoroscopy provided detailed information on lumbar segmental motion characteristics (MSI = motion sharing inequality) in recumbent passive flexion [21]. However, the use of special setup and ionizing radiation limits its use in daily clinical practice. Lordosis distribution index (LDI), introduced by Yilgor et al. [22], defines the magnitude of lower arc lordosis relative to the total lordosis. The LDI is described as the L4-S1 lordosis divided by the L1-S1 lordosis and multiplied by 100. However, it has limited use in describing variation in upper lumbar and L4-L5 and L5-S1 lordosis. Frobin et al. [23] published a detailed and well-documented article on spinal geometry. They proposed an alternative method using lateral lumbar spine X-ray precision measurement of the disc and vertebral body height and sagittal plane displacement (antero- and retrolisthesis). The extensive dataset on normal, age-adjusted values of the abovementioned parameters can be used to compare individual case quantitative assessment. However, in our study, we used MRI scans and selected different parameters of interest.

The MRI is the most widely used technique of evaluating lumbar intervertebral disc degeneration. The normal intervertebral discs show sharp borders between nucleus pulposus and annulus fibrosus on T2-weighted MRIs because of the signal brightness. Furthermore, intervertebral disc degeneration shows a reduction in signal. In the literature, many grading systems are used to classify the intervertebral disc degeneration, e.g., the grading system of Pfirrmann et al. [24] or the modified Pfirrmann grading system by Griffith et al. [25]. Pfirrmann grading system classifies disc degeneration using criteria of disc structure, the distinction of nucleus and annulus, signal intensity, and disc height into 5 grades. Modified Pfirrmann grading system uses 8 grades for categorization in the older population. There is a thorough review of recommended grading systems for lumbar disc degeneration [26].

The aim of this study was to develop a method to assess the local distribution of total lumbar lordosis selecting an optimal, single quantitative parameter which is reliable and it can be calculated easily with a free computer software in the everyday clinical practice. Furthermore, this parameter was linked to lumbar intervertebral disc degeneration using standard MRIs.

## 2. Materials and Methods

### 2.1. Lagrange Polynomial Approximation for Lumbar Lordosis Curvature

**Figure 1:**
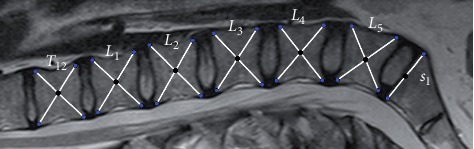
Mark of the vertebrae corners with the Spinalyze Software.

On each image, the outermost upper and lower corners of the five lumbar and the T12 vertebrae and also the upper corners of the S1 vertebral bodies were selected. The selected points defined five vertebral centers (i.e., centroids) and one upper-middle point for the sacrum (Figure 1).

The shape of the spine was investigated with a mathematical approximation method where the unknown values of a function were approximated based on known values. Lagrange interpolation was used to investigate the line because this method was found to be the most appropriate one and easy to interpret among professionals. The steps of our method are as follows:

1. Let *P*_0_ (0, 0) be the measured center of the T12 thoracic vertebra.
2. Let *P*_*i*_ (*x*_i_, *y*_i_) be the measured center of Li lumbar vertebrae (*i* = 1, 2, 3, 4, 5).
3. Let *P*_6_ (*x*_6_, *y*_6_) = *P*_6_ (*x*_6_, 0) be the measured point of sacrum, where the second coordinate is zero (the *x*-axis is *P*_0_ (0, 0) and *P*_6_ (*x*_6_, 0)).
4. The spine is located above the *x*-axis.
5. Let the interpolation polynomial in the interval [*x*_0_, *x*_6_] be as follows:

(1) $$\begin{array}{c} p\left(x\right)=\sum_{i=0}^{6}y_{i}L_{i}\left(x\right), \end{array}$$

where the function of Lagrange interpolation is

(2) $$\begin{array}{c} L_{i}\left(x\right)=\underset{k\neq i}{\prod_{k=0}^{6}}\frac{x-x_{k}}{x_{i}-x_{k}}. \end{array}$$

**Figure 2:**
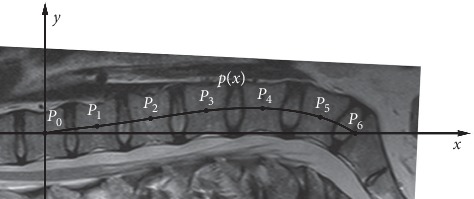
The Lagrange interpolation polynomial to approximate the curvature of lumbar lordosis.

The essence of the procedure is to use the measured centers of the vertebrae to approach the line of the spine with a polynomial that provides a much finer approximation than a simple arc or ellipse. With Lagrange interpolation, just one such interpolation polynomial can be aligned on the spine line; that is, the polynomial is unique. In addition, the polynomial is a continuous, differentiable, and integrable function, and at the examined interval, the function takes up the maximum and the minimum (Figure 2).

### 2.2. New Parameters for Characterizing the Lumbar Lordosis Distribution

**Figure 3:**
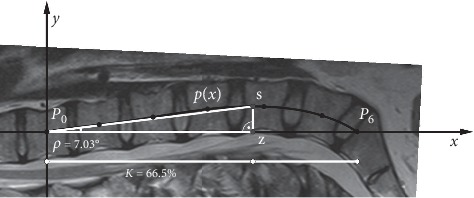
Determination of Rho-angle and digression percentage.

Based on the polynomial, new parameters were defined to discover the local behavior of lumbar lordosis. The aim of the parameters is that they describe the deflection, the location of the maximum deflection, and the expansion of the lumbar spine. These parameters are as follows:

- Rho-angle (*ρ* angle): let S (*x*_S_, *y*_S_) be the maximum of the *p*(*x*) polynomial on the [*x*_0_, *x*_6_] interval. Let Z (*x*_S_, 0) be the orthogonal projection of the S point onto the *x*-axis. Let Rho-angle (*ρ* angle) be the Z*P*_0_S angle in the *P*_0_SZ right-angled triangle. Graphically, the Rho-angle gives, in this section, the maximum deflection angle of the lumbar lordosis from the *x*-axis (T12—sacrum) (Figure 3).
- Digression percentage (*K*%): let digression percentage (*K*%) be

(3) $$\begin{array}{c} K=\frac{x_{S}}{x_{6}}100. \end{array}$$

  Graphically, the digression percentage gives the location of the maximum deflection in this section (T12—sacrum) (Figure 3).
(c) Expansion percentages (A_1_, A_2_, A_3_, A_4_, A_5_, A_6_ %s): let expansion percentages be

(4) $$\begin{array}{c} A_{i}=\frac{T_{i}}{T},\;\left(i=1,2,3,4,5,6\right), \end{array}$$

where

(5) $$\begin{array}{c} T_{i}=\int_{x_{i-1}}^{x_{i}}p\left(x\right)\text{d}x\\ T=\int_{x_{0}}^{x_{6}}p\left(x\right)\text{d}x. \end{array}$$

**Figure 4:**
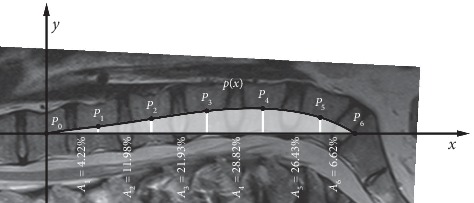
Determination of expansion percentages.

Graphically, the expansion percentages give, in this section, the proportion of the local expansion (between the middles of two vertebrae) and the global expansion (between middles of T12 and sacrum) (Figure 4).

### 2.3. Software Availability and Functions

MRIs were analysed with a personal computer and our software which is based on GeoGebra software. GeoGebra (https://www.geogebra.org/) is a dynamic mathematics software package for all levels of education that brings together geometry, algebra, spreadsheets, graphing, statistics, and calculus in one easy-to-use package.

**Figure 5:**
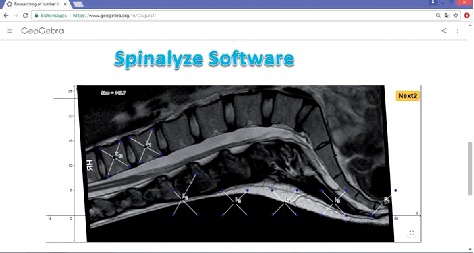
Spinalyze Software on GeoGebraTube.

Thanks to the development of technology, the demand for IT tools has increased and there are many articles where the researchers use smartphones and applications for global spine measurement [27]. Therefore, we wanted to develop software that is free and easy to use on a daily basis. The name of our method is the SRD-method. (Sandor–Rathonyi–Dinya method) and the name of our computer program is Spinalyze Software. Spinalyze Software is browser based (anyone can use it with a web browser) and available for free worldwide: http://www.spinalyzesoftware.com. There is a website on GeoGebraTube which contains introduction information, the Spinalyze Software (with anonymous sample MRI pictures), a user's guide (in YouTube video), and a feedback form (in Google Form) (Figure 5). The Spinalyze Software calculates the approximating polynomial, the Cobb-angle, and the new parameters.

### 2.4. Subject Data

Patients were randomly sampled and received outpatient care for low back pain. In our research, the MRIs of 60 patients (21 male and 39 female persons with different lumbar problems) were analysed with our software. All subjects with major spinal deformities, chronic inflammatory conditions, previous history of a spinal tumor, infection, trauma, or surgery were excluded, and all patients had chronic mechanical local low back pain treated with nonoperative measures. Approval from the local Research Ethics Committee had been obtained and all anonymised images used in this study had been taken for clinical diagnosis previously at our institution. MRIs were taken with various scans. However, in each case, only one T2-weighted FSE midsagittal image was included by selecting the one with the widest spinal canal and spinal process of the sagittal series.

### 2.5. Statistical Analysis

The *p* value < 0.05 was considered statistically significant, and a two-sided test was applied. The analyses were done with IBM SPSS Statistics 25.0 (SPSS, Chicago, IL).

#### 2.5.1. Reliability Analysis and Measurement Error of the Spinalyze Software

The first step is loading the MRIs into the software, followed by manually selecting the measurement points. Designating the measurement points is an essential aspect because it determines the quality of our metric. Each one of two independent observers marked the measurement points twice with an interval of 5 days. The coordinates of the points provided by the software were recorded in an MS Excel database, and the consistency and reliability of the two readings were evaluated with SPSS. In the reliability analysis, intraclass correlation coefficients (ICCs) were calculated to determine the intraobserver reliabilities. ICC(2, 1) estimates and their 95% confidence intervals were calculated based on a single rater/measurement, absolute-agreement, 2-way random effects model [28, 29]. According to Winer [30], the ICC was rated as follows: 0 to 0.24, weak; 0.25 to 0.49, low; 0.50 to 0.69, average; 0.70 to 0.89, good; and 0.90 to 1, excellent. The consistency of the two readings of the observers was investigated by interclass correlation (by Pearson's *r* coefficients). There are many rules that suggest the correlation for the absolute value of *r*, among which the rule by Evans is widely used [31]: 0 to 0.19, very weak; 0.20 to 0.39, weaker; 0.40 to 0.59, significant; 0.60 to 0.79, strong; and 0.80 to 1, very strong correlation. The measurement error (or repeatability) was calculated as 2.77 times the within-subject standard deviation [32] as determined using a one-way analysis of variance.

#### 2.5.2. Descriptive Statistics of Sample and the New Parameters per Gender

The data of patients (age, body height, body weight, and body mass index (BMI)) were evaluated and their major parameters were determined. The normality distribution of the variables was checked by the Shapiro–Wilk W test. For comparison between gender groups, an independent two-sample *t*-test was used, and Cohen's *d* value was calculated to express the effect size. Where normality was not met, the nonparametric version of the independent two-sample *t*-test, the Mann–Whitney *U* test, was used. For the new parameters, each of the 60 images was measured twice by two observers and the measured four values were averaged. Cobb-angle was calculated between L1 superior endplate and L5 inferior endplate.

#### 2.5.3. Reliability Analysis of the Grading by Pfirrmann Grading System

Two observers, with different levels of experience analysing spinal MRIs, independently graded the 300 lumbar intervertebral discs (from the 60 patients), using the 5-level Pfirrmann grading system [24]. The system was devised from asymptomatic subject cohort with a mean age of 40 years (range, 10–83 years), and the modified Pfirrmann grading system was improved to an older population with a mean age of 73 years (range, 67–83 years) [25]. The reasons for choosing the Pfirrmann grading system were that this useful grading system has been accepted and applied clinically [33–35], and our population was with a mean age of 44.22 years (range, 15–78 years). All discs were graded in a single session. The reliability of the MRI evaluations was estimated using agreement percentage and Cohen's kappa statistics between observers (interobserver reliability) [36]. According to Landis and Koch [37], the agreement was rated as follows: kappa 0 to 0.20 indicated slight agreement; 0.21 to 0.40, fair agreement; 0.41 to 0.60, moderate agreement; 0.61 to 0.80, substantial agreement; and 0.81 upward, excellent agreement. With this rating, the absolute agreement would be 1.

#### 2.5.4. Relationships Analysis

In the correlation analysis, Pearson's *r* value was used to express the intensity of the relationship between the variables. Where the normality criterion was not met, the nonparametric Spearman's correlation value procedure was used. In the first analysis, the relationships were examined between the MRI morphological parameters (gold standard Cobb-angle, our new parameters). In the second analysis, the relationships were investigated between the Pfirrmann grading system categories of L1-S1 lumbar discs and the MRI morphological parameters. Observer 1 reading results were used in the calculation, as he was the most experienced investigator.

## 3. Results

### 3.1. Reliability Analysis and Measurement Error of the Spinalyze Software

The intraobserver reliability for all measurement points was found in the excellent category (ICCs > 0.90). In the interobserver reliability, the Pearson *r* values were significant for each measurement point and they were in the very strong correlation category (values were *r* > 0.90). The average within-subject standard deviation on the model was 0.07 SD for the first observer and 0.20 SD for the second observer. The average measurement error on the model was 0.19 for the first observer and 0.57 for the second observer.

### 3.2. Descriptive Statistics of Sample

Significance of males and females with mean ± standard deviation was as follows: age (41.7 years ± 10.9; 45.6 years ± 15.8; *p*=0.273), body height (179.6 cm ± 6.8; 166.3 cm ± 8.5; *p* < 0.001, Cohen's *d* = 1.73), body weight (84.9 kg ± 9.8; 67.6 kg ± 12.6; *p* < 0.001, Cohen's *d* = 1.53), and BMI (26.3 ± 2.8; 24.6 ± 4.9; *p*=0.092). Significant differences were found only in body height and body weight, while there was no significant gender difference in age and BMI.

### 3.3. Investigation of MRI Morphological Parameters

#### 3.3.1. Descriptive Statistics of the New Parameters per Gender

Table 1 shows the descriptive statistics of the new morphological parameters for gender. Among them, Cobb-angle and the A_5_ segment have the largest variability, and the Rho-angle and A_6_ segment are the least variables. The variables were compared among genders and no significant difference was found.

#### 3.3.2. Relationships of Parameters

Only the Cobb-angle and A6 were normally distributed. The correlation values are in Table 2.

The main results of Table 2 are as follows. (1) With the Cobb-angle: (i) there is a strong linear relationship with the Rho-angle (*rϱ* = 0.9374), (ii) digression % does not show a statistical relationship, and (iii) relationships with A_1_-A_2_-A_3_ have a positive correlation. (2) Examining the correlation of the other variables one by one: (i) Rho-angle has a medium positive relationship with A_3_ and is negatively related to A_5_, and (ii) digression % has a strong negative relation with A_2_-A_3_ and good positive relation with A_5_.

#### 3.3.3. Reliability Analysis of the Grading by Pfirrmann Grading System

Applying the Pfirrmann grading system, the number of disc degeneration grades assessed by each observer is summarized in Table 3. The interobserver reliability of grading yielded 88.33% agreement percentage and kappa value of 0.84 (with SE of kappa = 0.025, 95% confidence interval: from 0.791 to 0.890), which was in the excellent agreement category. The weighted kappa was 0.896.

#### 3.3.4. Relationship between Pfirrmann Grades and MRI Morphological Parameters

**Figure 6:**
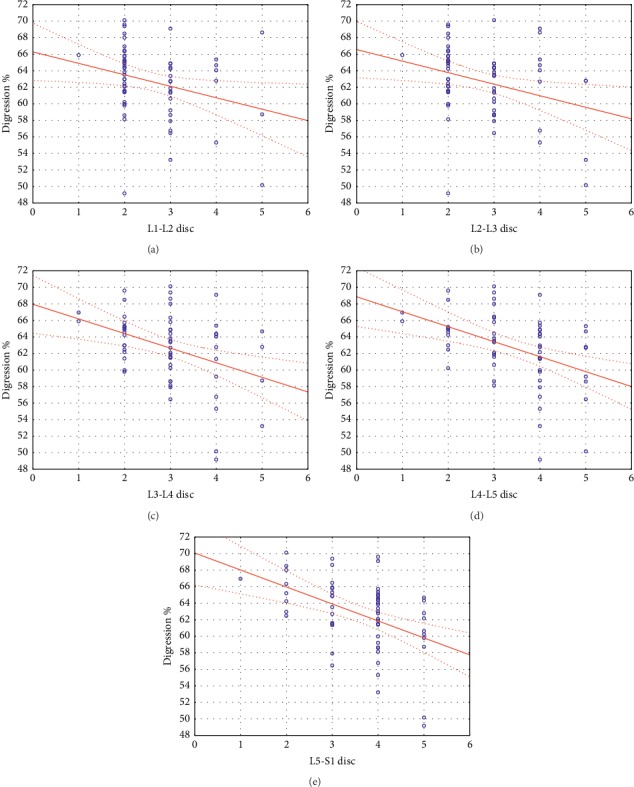
The relationship between lumbar disc grades and digression percentage (*K*%).

We found that for Cobb- and Rho-angles only L4-L5 disc grade values showed a significant relationship with both angles (*rϱ* of 0.2825 and 0.3876, respectively), whereas digression percentage (*K*%) showed a significant negative correlation with all L1-S1 disc grades with increasing *r* values (*rϱ*_1_ = −0.2880, *rϱ*_2_ = −0.2814, *rϱ*_3_ = −0.3534, *rϱ*_4_ = −0.4395, and *rϱ*_5_ = −0.4582).  Figure 6 illustrates the relationship between lumbar disc grades and digression percentage (*K*%). The expansion percentages did not show relationships.

## 4. Discussion

The aim of this study was to develop a reliable new method to assess the local distribution of total lumbar lordosis with a single numeric parameter using standard MRIs and find a correlation with lumbar intervertebral disc degeneration.

In our research, we analysed MRIs of 60 lumbar patients (21 male and 39 female persons) with our new Spinalyze Software based on a novel mathematical approach. The intra- and interobserver reliability (expressed as the ICC and the Pearson *r* value) and measurement error analysis used in our study were similar to those found by other studies [18, 38–40]. The ICCs were in the excellent category (ICCs > 0.90) and the Pearson *r* values were in the very strong correlation category (*r* > 0.90). The results confirm that the consistency between the two readings of each observer is maximized, similar to the safety of reading between two observers [41]. Determining the measurement points can be done easily and safely, so the parameters obtained by further calculations will be reliable.

**Figure 7:**
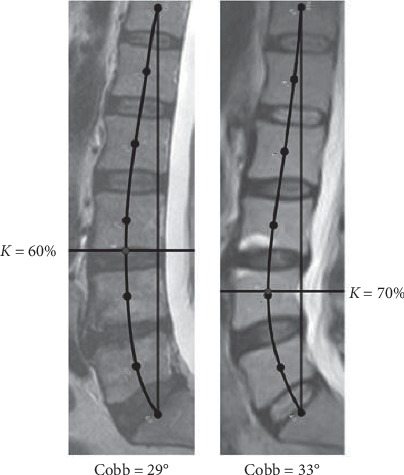
Different digression percentages (*K*%s) with similar Cobb-angles.

To describe the segmental distribution of lumbar lordosis, we developed new parameters: Rho-angle, digression percentage (*K*%), and expansion percentages. The parameters were compared among genders, and we did not find any significant difference. Based on our results, these parameters were independent of gender. The correlations were investigated between MRI morphological parameters. According to our findings, there was a strong linear relationship between the Cobb-angle and the Rho-angle (*rϱ*  = 0.9374). The *K*%, the digression percentage, gives the location of the maximum deflection in this section (T12 and sacrum). Based on our results, the *K*% was with a mean value of 62.68% (with ±4.36% std. Dev.) which is away from the 50% middle position; moreover, the digression percentage did not show a statistical relationship with the Cobb-angle. In Figure 7, we can see two spines with similar Cobb-angles but big difference digression percentages. The Cobb-angle describes the spine with a single, even arc, but it is not a precise approximation of the spine. According to our results, the maximum deflection breaking point of lumbar lordosis and its location can be different with the same Cobb-angle. Consequently, there are spines with the same Cobb-angle but different local lumbar lordosis shapes (i.e., different segmental-local distribution of overall-global lordosis). This is in line with the great variability of segmental lordosis described by Meakin et al. [18]. The expansion percentages give the quantities (in percentages) of the lumbar lordosis expansion between the middles of two vertebrae. The sum of the A_4_-A_5_-A_6_ means, which is 58.79%, shows that the lumbar lordosis expansion focused between L3 center and sacrum. The Cobb-angle has a positive correlation with A_1_-A_2_-A_3_, no statistical relationship with A_4_, and a negative correlation with A_5_-A_6_. Therefore, it seems that the turning point is by A_4_. Consequently, the distribution of global lordosis is uneven because the shape of the lumbar lordosis is shifted downward and centered around the L4 lumbar vertebra, which is in line with other studies [10, 14]. Similar findings were published by Meakin et al. [18] using an active shape model.

The Pfirrmann grading system was used for the classification of lumbar intervertebral disc degeneration. The interobserver reliability (expressed as agreement percentage and kappa value) was investigated with two independent observers. The results in our study presented that the interobserver reliability was in the excellent agreement category (88.33% agreement percentage and kappa value of 0.84). Consequently, no obvious differences were seen between the two readers despite their different backgrounds and levels of experience. This is in line with the interobserver reliability analysis described by Pfirrmann et al. [24].

In the second correlation analysis, the relationships were investigated between the Pfirrmann grading system categories of L1-S1 lumbar discs and the MRI morphological parameters. According to our findings, only L4-L5 disc grade values showed a significant relationship with the Cobb-angle (*rϱ* = 0.2825) and Rho-angle (*rϱ* = 0.3876). Based on our results, digression percentage (*K*%) showed a significant negative correlation with all L1-S1 disc grades with increasing *r* values (*rϱ*_1_ = −0.2880, *rϱ*_2_ = −0.2814, *rϱ*_3_ = −0.3534, *rϱ*_4_ = −0.4395, and *rϱ*_5_ = −0.4582). This means that the smaller the value of digression percentage (*K*%), the more the number of worn discs in the lower lumbar sections (Figure 6). These results indicate that the more the total lordosis is concentrated in the lower segments, the fewer the number of the degenerated discs is in the lumbar spine. It was also true that digression percentage (*K*%) is a more sensitive parameter at Cobb- and Rho-angles to express the degree of degeneration. Consequently, based on our results, our new digression percentage (*K*%) parameter was linked to lumbar intervertebral disc degeneration using standard MRIs.

Our study has some limitations. First, other software programs exist for spinal examination (e.g., Surgimap) but we wanted to implement our SRD-method with new morphological parameters and to develop a free, reliable, easy-to-use, online, open-source software for daily clinical use. Furthermore, our study includes a somewhat limited sample size. However, similar studies worked with comparable or lower (24 or 37) samples [18, 21, 24, 25]. Moreover, limited number of measurement points selected manually (i.e., 4 corners) could adversely affect precision [18]. We plan to overcome this problem later by using AI (artificial intelligence) image processing. We would like to improve the Spinalyze Software with new modules and to create a database where we can save and analyse anonymous medical images from all over the world. Second, we are aware that other studies using the standing position could give different geometry but we chose supine scans as they used in everyday clinical practice. The extension of this study to larger case numbers in a multicentric setting seems only possible using regular MRI scans and the degree of disc degeneration does not depend on the MRI scan position. Furthermore, all MRIs were acquired for clinical diagnosis with different scans at various institutions. However, this will be the case when our new method will be used online with multicentric input for further connection investigations among local distributions of global lordosis and disc degenerative changes and symptoms (pain, dysfunctions, etc.). The impact of information regarding the connection between spinal geometry and pathology is substantial not only in surgical reconstruction but also in postural-based physical rehabilitation.

## 5. Conclusion

In conclusion, the new Spinalyze Software based on a novel mathematical approach provides a free, easy-to-use, reliable, and online measurement tool using standard MRIs to approximate the curvature of lumbar lordosis. The new reliable *K*% (digression percentage) is one single quantitative parameter to assess the local distribution of total lumbar lordosis. Based on our results, the smaller the value of *K*%, the more the number of worn discs in the lower lumbar sections. These results indicate that digression percentage (*K*%) may possibly be associated with the development of lumbar intervertebral disc degeneration. Further evaluation is needed to assess its behavior and advantage.

## Figures and Tables

**Table 1:** Descriptive statistics of the morphological parameters.

Variables	Gender	*N*	Mean	Std. deviation	Gender total		
					*N*	Mean	Std. deviation
Cobb-angle	Female	39	33.44	12.19	60	33.43	12.12
	Male	21	33.41	12.27			

Rho-angle	Female	39	7.74	3.11	60	7.80	2.97
	Male	21	7.90	2.76			

Digression %	Female	39	63.15	4.11	60	62.68	4.36
	Male	21	61.80	4.77			

A_1_	Female	39	3.07	6.68	60	3.37	5.50
	Male	21	3.93	1.95			

A_2_	Female	39	13.86	6.65	60	13.95	5.94
	Male	21	14.13	4.50			

A_3_	Female	39	23.36	5.44	60	23.89	4.81
	Male	21	24.88	3.24			

A_4_	Female	39	29.99	3.92	60	29.97	3.40
	Male	21	29.94	2.21			

A_5_	Female	39	24.86	10.25	60	23.99	8.93
	Male	21	22.36	5.61			

A_6_	Female	39	4.87	2.14	60	4.83	2.15
	Male	21	4.76	2.24			

**Table 2:** Correlation values among parameters.

Variables	Spearman correlations								
	Cobb-angle	Rho-angle	Digression (%)	A_1_	A_2_	A_3_	A_4_	A_5_	A_6_
Cobb-angle	1.0000								
Rho-angle	0.9374^*∗*^	1.0000							
Digression %	−0.1817	−0.3153^*∗*^	1.0000						
A_1_	0.2717^*∗*^	0.3398^*∗*^	−0.6051^*∗*^	1.0000					
A_2_	0.3484^*∗*^	0.4457^*∗*^	−0.7130^*∗*^	0.7106^*∗*^	1.0000				
A_3_	0.4748^*∗*^	0.5722^*∗*^	−0.6898^*∗*^	0.7293^*∗*^	0.7862^*∗*^	1.0000			
A_4_	−0.0785	−0.1571	0.5859^*∗*^	−0.5700^*∗*^	−0.6027^*∗*^	−0.3497^*∗*^	1.0000		
A_5_	−0.4254^*∗*^	−0.5298^*∗*^	0.6742^*∗*^	−0.8252^*∗*^	−0.8318^*∗*^	−0.9537^*∗*^	0.3673^*∗*^	1.0000	
A_6_	−0.4302^*∗*^	−0.4898^*∗*^	0.4868^*∗*^	−0.6297^*∗*^	−0.7623^*∗*^	−0.8397^*∗*^	0.1356	0.8523^*∗*^	1.0000

*Notes.* The correlation matrix is symmetrical to the main diagonal, so it is sufficient to display the values of the lower triangle. ^*∗*^Correlations are significant at *p* < 0.05.

**Table 3:** The number of disc degeneration grades assessed by each observer (Gi : Pfirrmann's grades).

		Observer 2					Total
		G1	G2	G3	G4	G5	
Observer 1	G1	6	1	0	0	0	7
	G2	0	83	12	0	0	95
	G3	0	6	84	5	0	95
	G4	0	0	1	66	7	74
	G5	0	0	0	3	26	29
	Total	6	90	97	74	33	300

## Data Availability

The MRI data used to support the findings of this study are available from the corresponding author upon request.

## Abbreviations

MRI: Magnetic resonance imaging
T12: 12th thoracic vertebra
L1, L2, L3, L4, L5: 1st, 2nd, 3rd, 4th, 5th lumbar vertebra
S1: Sacrum first segment
*P*_0_: Measured center of the T12 thoracic vertebra
*P*_1_, *P*_2_, *P*_3_, *P*_4_, *P*_5_: Measured center of Li lumbar vertebrae (*i* = 1,…, 5)
*P*_6_: Measured upper middle point of sacrum
*p*(*x*): Interpolation polynomial
L_i_(*x*): Function of Lagrange interpolation
S: Maximum of the *p*(*x*) polynomial on [*x*_0_, *x*_6_] interval
Z: Orthogonal projection of the S point onto the *x*-axis
*ρ*: Rho-angle (degree)
K: Digression percentage (%)
A_1_, A_2_, A_3_, A_4_, A_5_, A_6_: Expansion percentages (%)
ICC: Intraclass correlation coefficient
BMI: Body mass index
r: Pearson's correlation coefficient
r*ϱ*: Spearman's correlation coefficient
G1, G2, G3, G4, G5: Pfirrmann's grades
L1-L2 disc: Disc between L1 and L2 lumbar vertebrae.
